# Simulation-Based Virtual-Reality Patient-Specific Rehearsal Prior to Endovascular Procedures: A Systematic Review

**DOI:** 10.3390/diagnostics10070500

**Published:** 2020-07-20

**Authors:** Caroline Albrecht-Beste Nielsen, Lars Lönn, Lars Konge, Mikkel Taudorf

**Affiliations:** 1 Department of Radiology, Rigshospitalet, 2100 Copenhagen, Denmark; Lars.birger.loenn@regionh.dk (L.L.); Mikkel.Taudorf@regionh.dk (M.T.); 2 Faculty of Health and Medical Sciences, University of Copenhagen, 2200 Copenhagen, Denmark; 3 Copenhagen Academy for Medical Education and Simulation (CAMES), Capital Region of Denmark, 2100 Copenhagen, Denmark; lars.konge@regionh.dk

**Keywords:** simulation, training, assessment, clinical competence, systematic review, endovascular, patient-specific rehearsal

## Abstract

Patient-specific rehearsal (PsR) is a new concept whereby a procedure can be rehearsed virtually using the exact patient’s anatomical data prior to performing the real procedure. The aim of this study was to ascertain if endovascular virtual-reality PsR simulation enhanced performance in real life. This was done by performing a systematic review following the preferred reporting items for systematic reviews and meta-analysis (PRISMA) guidelines. A literature search was conducted in PubMed, Embase, The Cochrane Library and Web of Science concerning PsR in endovascular procedures. All publications were handled using Covidence. Reference lists were also screened. Data extracted from the studies were realism rating, procedure time, fluoroscopy time, contrast volume, number of angiograms and reduction of errors. Kirkpatrick’s four-level model for measuring the efficiency of training was used for guidance of the relevance of studies. The search yielded 1155 results after the exclusion of duplicates, and 11 studies were included. Four studies had a control group, including one randomized trial; the remaining seven were feasibility studies. The realism was rated high, and overall, the studies showed improvements in procedure time, fluoroscopy time and contrast volume after PsR. One study assessed and confirmed the reduction in errors after PsR. Only two studies included more than 15 patients in their cohort. Kirkpatrick’s model was applied to all studies, with one study reaching level 4. All studies found the concept of PsR to be feasible and realistic. The studies with a control group showed a reduction of overall procedure time, radiation exposure and potential errors in endovascular procedures following PsR.

## 1. Introduction

Simulation is deeply rooted in the aviation industry, where it is used in pilot assessment and certification [1,2]. All pilots undergo training and recertification in a simulator every six months, where they are exposed to a wide range of flight conditions [1]. The aerospace manufacturer’s training is highly specific to the actual planes that the pilots will fly when certified and includes training on the specific routes and airports used [1]. Inspired by aviation, simulation is incorporated into the healthcare system as a tool to improve skills and increase patient safety [3]. The benefits of virtual reality simulation training are clearly demonstrated in several surgical fields, but as of yet, these simulations have been based on generic cases rather than focusing on the specific patient [4,5,6,7]. It is important to remember that simulation training cannot replace the traditional apprenticeship model completely but can be more efficient [8,9], and patient-specific simulation might further improve the efficacy of training. 

From a simulated case of an existing patient’s anatomy, a concept labeled “patient-specific rehearsal” (PsR), “case-specific” rehearsal or “mission” rehearsal is created. In virtual-reality (VR) PsR, digital imaging and communications in medicine (DICOM) data from a computed tomography angiography (CTA) or a magnetic resonance angiography (MRA) scan with the relevant anatomy are reconstructed using a segmentation solution. The reconstruction is imported to the simulator using a standard triangle language (STL) file, and the technology creates a vascular “twin” anatomy for procedural training, i.e., a virtual 3D model of the anatomy. Endovascular surgery, as compared with other surgical fields, has been the first to build a model of a specific case on which to operate. The model can be used to share and teach before, during and after the actual procedure. Ultimately, it can be used as a rehearsal or a warm-up before the actual performance in the angio suite.

The purpose of PsR is to prepare for the procedure with the surgical team to potentially foresee problems, reduce exposure to radiation, shorten procedure times, reduce equipment use and increase patient safety [10]. Endovascular procedures such as carotid artery stenting (CAS) and endovascular aneurysm repair (EVAR) are obvious cases to incorporate in PsR, as the setup is easy to reproduce. The need exists to investigate the technology of PsR and assess the advantages and possibilities in such a strategy.

The aim of this systematic preferred reporting items for systematic reviews and meta-analysis (PRISMA) review was to examine whether VR PsR can improve operator skills and techniques and provide an overview of the published literature to evaluate the benefits of the technique in endovascular procedures. To our knowledge, this is the first review that explores PsR connected to endovascular procedures.

## 2. Materials and Methods 

This systematic review was designed according to the preferred reporting items for systematic reviews and meta-analysis (PRISMA) guidelines [11]. 

### 2.1. Search Strategy

The literature search was conducted on 18 March 2020, and was performed in PubMed, Embase, the Cochrane Library and Web of Science. The following search strings were used:

Search string 1: (“Patient-specific*” OR “Patient-based*” OR “Patient-specific modeling [MeSH]”) AND (“rehearsal*” OR “simulat*” OR “Computer simulation [MeSH]”) AND (“Endovascular*” OR “Intravascular*” OR “Vascular* OR “Endovascular procedures [MeSH]”).

Search string 2: (“Case-specific*” OR “Case-based*”) AND (“Endovascular*” OR “Intravascular*” OR “Vascular*”).

The asterisk included words with different suffixes in the search. The search strings were developed in collaboration with an experienced research librarian. 

### 2.2. Selection Process

All studies on the patient-specific rehearsal simulation-based training of endovascular procedures were eligible. Inclusion criteria were original peer-reviewed publications concerning medical professionals on patient-specific or case-specific endovascular procedure, simulation, training or rehearsal, and simulations were to be followed by an operation. Exclusion criteria were publications concerning 3D printing, glass models, silicone models and foam models. There was no limitation on publication year. 

All search results were managed in the online Cochrane technology platform, Covidence [12]. 

After the removal of duplicates, all articles were screened by titles and abstracts by two authors (CN and MT), and non-relevant studies were excluded.

Reference lists of included studies were additionally screened, and a search for papers citing the included studies was conducted in Web of Science to identify other potentially eligible publications, and none were found. 

### 2.3. Data Collection

Data extracted from the included articles were procedure, participants, number of patients, procedure time, fluoroscopy time, contrast volume, number of angiograms, errors, technical success rates, subjective assessment, where the simulation was conducted, changes in initial plans, fluoroscopy and C-arm angulations, procedure equipment and material comparison, technical ratings on the global rating scale (GRS) or procedure-specific rating scale (PSRS) and non-technical (human factor) ratings on the non-technical skills for the surgeon rating scale (NOTECHS) or the Mayo High Performance Team Scale (MHPTS) [13]. 

### 2.4. Kirkpatrick’s Model Designed to Objectively Measure the Effectiveness of Training

Analysis of all studies were guided by Kirkpatrick’s model [14], which describes four different levels on which to measure the efficacy of training. 

Level 1: Reaction—evaluates participants’ subjective assessment of how realistic, useful, etc., the simulation training was found to be. 

Level 2: Learning—evaluates the effect of the simulation training in a simulated environment, determining whether the training resulted in improved performance on the simulator used in training or on any other simulator. 

Level 3: Behavior—evaluates the effect of simulation training on the participants’ behavior or performance in real life. This can be measured by, for example, procedure time, fluoroscopy time, contrast volume and number of angiograms.

Level 4: Results—evaluates the effect of the simulation training on patient outcomes, measured by, for example, mortality rate and complications [14].

### 2.5. Bias Assessment

Bias assessment was conducted by two authors (CN and MT) using the Medical Education Research Study Quality Instrument (MERSQI) [15]. In this assessment the studies are evaluated in 6 domains (study design, sampling, type of data, validity evidence, data analysis and outcome) with a potential maximum total score of 18.

## 3. Results

### 3.1. Study Selection

The search was conducted on 18 March 2020 and resulted in 2022 studies, of which 867 duplicates were excluded. In all, 1155 studies were subsequently screened, and of these, 1134 studies did not meet inclusion criteria based on title and abstract. The full texts of 21 articles were screened by the same two authors, and a consensus was reached, resulting in the 11 articles included in this review.

**Figure 1:**
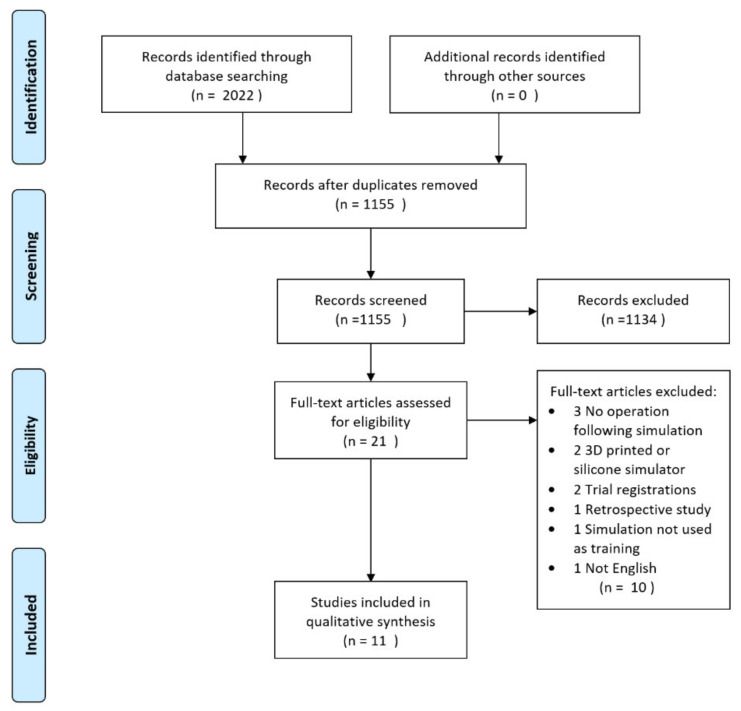
Preferred reporting items for systematic reviews and meta-analysis (PRISMA) flowchart.

Data extraction was completed by the same two authors, and discrepancies were resolved through collaborative decision-making. The study selection scheme is summarized in Figure 1. 

Of the 11 included articles, 5 articles described PsR in carotid artery stenting (CAS), 5 involved endovascular aortic aneurism repair (EVAR), and finally, 1 involved endovascular thoracic aortic repair (TEVAR). The 11 studies were divided into 2 groups: studies with a control group, shown in Table 1, and feasibility studies, shown in Table 2.

### 3.2. Study Characteristics 

In the four studies with a control group, metrics were compared between two patient groups: one with PsR prior to the procedure and one without PsR. The results are listed in Table 1. The two papers by Desender et al. (2016) [16] and (2017) [13] were based on the same cohort. This was the only randomized control trial in this review, although Wooster et al. [17] actively randomized the enrollees by computer settings. In Våpenstad et al. [18], the patients were allocated to the PsR group when a simulator was available. In Wooster et al. [17], the training location changed during the trial, and the study was thus terminated prematurely, which the authors stated affected the p-values, all of which were >0.05. 

The seven feasibility studies evaluated at level 1 in Kirkpatrick’s model are listed in Table 2. Here, a PsR simulation was completed 24 h prior to the actual procedure, and the metrics from the simulation were compared with the metrics from the actual procedure.

Ten studies used the ANGIO mentor (Simbionix, Cleveland, OH, USA), and one study, Våpenstad et al. [18], used the Vascular Intervention System Trainer (VIST) simulator (Mentice AB, Gothenburg, Sweden). 

The context for PsR differed between the studies. Willaert et al. (2010) [21] and (2012) [22] mentioned that some rehearsals were carried out in a real angiosuite, while Roguin et al. [20] used a seminar room, Desender et al. (2013) [23] used a laboratory, and finally, Pakeliani et al. [24] employed an office environment, with no resemblance to a real operating environment. 

Procedure time, fluoroscopy time and contrast volume were reported in the majority of the studies (Table 2). One study reached Kirkpatrick’s level 4, two studies achieved level 3 (see Table 1), and the remaining studies were level 1 (see Table 2).

### 3.3. Study Findings

Desender et al. (2016) [16] and (2017) [13] were the only studies to report patient outcomes and were therefore a level 4 in Kirkpatrick’s model. The in-house hospital mortality rate was 0% with the PsR group versus 2% in the control group, and 30-day mortality was 4% in the PsR group versus 2% in the control group (*p* = 1.00). This study also noted a significant reduction in minor and major errors, as well as issues that caused procedure delays, with changes of −26% (*p* = 0.004), −76% (*p* = 0.009) and −27% (*p* = 0.007), respectively.

**Figure 2:**
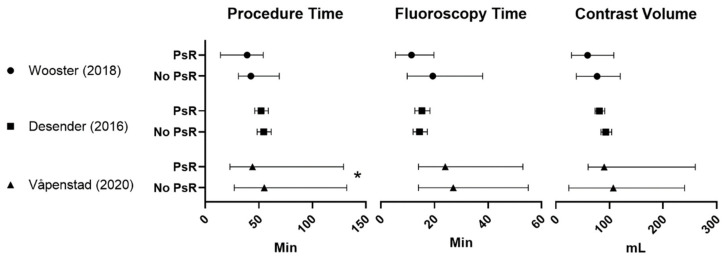
Studies with a control group comparison of procedure time, fluoroscopy time and contrast volume. Mean or median and range are given in Table 1. Min = minutes, mL = milliliter. * Significant *p*-value (*p* < 0.05).

All the studies with a control group, as depicted in Figure 2, reached level 3. The only study to show a significant reduction in overall procedure time for PsR was Våpenstad et al. [18] (*p* = 0.017) (Figure 2). However, both Wooster et al. [17] and Desender et al. (2016) [16] noticed a non-significant reduction in procedure time.

Desender et al. (2016) [16] noted a significant difference in the number of angiograms performed to visualize EVAR’s proximal and distal landing zones with differences of −23% (*p* = 0.005) and −21% (*p* = 0.004), respectively, with PsR. These metrics were, however, not evaluated in Våpenstad et al. [18] or Wooster et al. [17].

**Figure 3:**
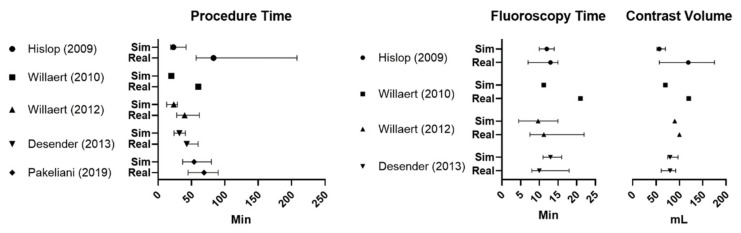
Feasibility study comparison of procedure time, fluoroscopy time and contrast volume. Mean or median and range or IQR (see Table 2 for details). Min = minutes, mL = milliliter.

A significantly shortened procedure time compared with the simulation was noted in Willaert et al. (2012) [22], Desender et al. (2013) [23] and Hislop et al. [19] (Figure 3); additionally, a reduced contrast volume and number of angiograms compared with the simulation were stated in one study each [19,22].

An altered treatment plan after PsR was noted in several studies. In Desender et al. (2017) [13], adjustments were made following the preplanning phase in 88% of the cases, and 92% of them were implemented in the real AngioLab procedure. In the work by Pakeliani et al. [24], a change was noted in 50% of the cases, and in Desender et al. (2013) [23], the PsR even resulted in the postponement of the procedure in one patient.

C-arm angulations to visualize the proximal and distal landing zone were altered in the preplanning phase after PsR in some of the studies. In Desender et al. (2013) [23], they were modified in 7/9 and 6/9 cases, respectively, and in Desender et al. (2017) [13], they were changed in 54% and 76% of the cases, respectively. 

The choice of material and tools in the procedure corresponded to the PsR, and in Willaert et al. (2012) [22], this was true in 11/15 of the cases. Surveys rated PsR as highly realistic, measured on a Likert or global rating scale [19,21,22,23]. 

The rehearsals had different settings and environments, including non-clinical settings, which certainly made comparing the studies difficult. However, the physical location did not affect the learning context, and site variance was considered to have minimal impact on the PsR results [26,27].

### 3.4. Bias Assessment

The MERSQI scores of studies with a control group are listed in Table 3, and those of the feasibility studies are in Table 4. The results show higher scores in the studies with a control group, indicating a lower risk of bias.

## 4. Discussion

This systematic review, conducted according to PRISMA standards, identified 11 studies on VR PsR before endovascular treatment in the angiosuite. One study found a reduction of potential errors with PsR [13,16]. Four studies with a control group showed a reduction in overall procedure time and fewer angiograms, with significant differences in one study for each, when using the PsR [13,16,17,18]. The papers differed in significant metrics; thus, a firm conclusion of overall benefit was a challenge. Wooster’s work [17] did not note a significant difference in metrics, which raises the question of a type II error, while Våpenstad’s [18] and Desender’s [16] work measured procedure time slightly differently, although this is presumably of minor importance. Våpenstad [18] measured from the first angiogram to the final angiogram, and Desender [16] instead measured from the introduction of the first guidewire to the removal of the final guidewire. Regardless of the type of simulator in the studies, the concept of PsR was demonstrated to be feasible and realistic [19,20,21,22,23,24,25]. The study design of the primary literature search differed greatly, yielding a small pool of included relevant studies. Nevertheless, the included studies pointed towards a positive effect of PsR-based training, with the potential to improve medical quality. 

A formal bias assessment was performed on all studies according to the MERSQI scale [15], i.e., an evaluation in 6 domains with a potential maximum score of 18. The studies with a control group scored better than the feasibility studies. Contributing to this was the fact that none of the seven feasibility studies were randomized, and the cohorts were limited [19,20,21,22,23,24,25]. Of the feasibility studies, the largest cohort, which was observed in Willaert et al. (2012) [22], had 15 patients enrolled. The Wooster group was a randomized study with a limited number of patients enrolled, thus increasing the bias risk in this study [17].

A recent review and meta-analysis explored the feasibility of patient-specific preoperative preparation in relation to all surgical specialties. This review included both virtual and physical models used for either patient-specific preoperative planning or rehearsal. This study concluded that PsR is safe and feasible in concordance with this review [28].

Future research is needed to determine the implications for implementation of PsR. To this point, the main shortcomings of the current studies were in (1) study design, (2) lack of power analysis and (3) outcome measurements, i.e., metrics according to acknowledged methods. First, randomized controlled trials (RCTs) are considered to be optimal studies, as the randomization minimizes bias, balances baseline characteristics and provides a basis for inference [29,30,31,32]. There was only one RCT included in this review, with the remaining studies using a control group randomized to some degree. Second, the cohort needs to be a certain size to ensure that the study is fully powered, for which different calculation tools exist [33,34]. Only two studies in this review exceeded 15 patients in their cohorts, and several studies did not result in significant p-values, i.e., underpowered study designs. Third, simulation can be used to assess competency, and several studies have demonstrated this in ultrasound procedures in radiology [35,36,37]. However, the assessment tools must be valid and, thus, careful background studies on assessment must be performed [38]. To link the Kirkpatrick’s model is undoubtedly warranted in a modern healthcare assessment strategy to assure the validity and clinical relevance and, thus, more high-end studies are needed to evolve PsR [39].

Standardized reporting of randomized studies supported by validation of assessment is warranted to reach the ultimate goal of transferring skills from the simulator context to the angiosuite.

Limitations of this Study

A limitation of this review is the small number of relevant articles. While simulation training with generic cases has been explored for a while, the use of patient-specific cases is a relatively new concept, and thus, few studies exist. Effectively, only a few data sets were available, showing that further studies need to be performed. Another limitation is the small cohorts and lack of power analysis with inherent difficulties in showing significant differences.

## 5. Conclusions

Endovascular simulation-based patient-specific rehearsal is a new area of research. This systematic review, conducted following the PRISMA guidelines, found the concept to be feasible and realistic. The studies showed a reduction in overall procedure time, radiation exposure and potential errors in endovascular procedures after rehearsal. The evidence level was low but in accordance with existing research within the broader simulation training context. Therefore, we conclude that this concept improves skills, but we cannot yet define the effect size.

## Figures and Tables

**Table 1:** Studies with a control group.

First author (No. of Perform. MDs)	No. of Patients	Procedure Time (min)	Fluoroscopy Time (min)	Contrast Volume (mL)	No. of Angiograms		Other End Points	Kirkpatrick’s Level
**CAS**								
Wooster (2018) [17] (3 MDs)	6 PsR	31.9 (14.2–54) +	11.4 (5.4–19.8)	59.2 (29–108) +	N/A			3
	9 controls	42.5 (30.9–69) +	19.4 (9.8–38) +	76.9 (38–120) +				
**EVAR**								
Desender (2016) [16] (99 MDs) ^a^ and Desender (2017) [13] ^b^	50 PsR	52.1 (46.2–58.8) +	15.3 (12.7–18.3) +	81 (73–91) +	6.5 (5.9–7.2) +	Proximal landing zone: −23% *^,^+ Distal landing zone: −21% *^,^+	Reduction in minor errors: −26% * Reduction in major errors: −76% * Reduction in errors causing delay: −27% *	4
	50 controls	54.6 (48.4–61.6) +	14.4 (12–17.3) +	93 (84–104) +	7.5 (6.7–8.2) +		Technical success rates: Primary: PsR 82% vs. control 78% Ass. Primary PsR 94% vs. control 86%	
Våpenstad (2020) [18] (16 MDs) ^c^	30 PsR	44 (23–129) *	24 (14–53)	90 (60–260)	7 (4–18)		Technical success rates: Primary: PsR 93% vs. control 77% Ass. Primary: PsR 3,3% vs. control 13%	3
	30 controls	55 (27–132)	27 (14–55)	107 (24–240)	7 (4–20)			

CAS = carotid artery stenting; EVAR = endovascular aortic aneurism repair; N/A = not available; PsR = patient-specific rehearsal; MD = Medical Doctor. All values are medians (range) unless otherwise stated. Metrics compare PsR vs. no PsR (control). * Statistically significant P-value (*p* < 0.05). + Mean values (range). ^a^ Sixty-eight percent of the procedures were performed by an experienced team, defined as a team in which at least 2 out of 3 members had performed at least 50 EVAR cases. ^b^ Desender et al. (2016) and Desender et al. (2017) are based on the same patient group, each article presenting different data. Green text represents Desender et al. (2017). ^c^ Five had less than 2 years’ experience, and the others had more than 2 years. There was at least one experienced operator present at each procedure.

**Table 2:** Feasibility studies.

First Author (No. of Perform. MDs)	No. of Patients	Procedure Time (min)	Fluoroscopy Time (min)	Contrast Volume (mL)	No. of Angiograms	Realism	Kirkpatrick’s Level
**CAS**							
Hislop (2009) [19] (3 MDs) ^d^	5	23 (19–42) vs. 83 (57–208) *	12 (10–14) vs. 13 (7–15)	57 (52–70) vs. 119 (57–175) *	N/A	GRS: 5 (4–5)	1
Roguin (2009) [20] (1 MD)	1	N/A	2.5 min <average	55 cc <average	N/A	N/A	1
Willaert (2010) [21] (2 MDs)	1	24.04 vs. 60.44	11.19 vs. 21.04	70 vs. 120	6 vs. 13	Likert: 4 (2–5)	1
Willaert (2012) [22] (3 MD) ^e^	15	23.75 (13–29) vs. 40.00 (28–62) *^,^^	9.70 (4.5–15) vs. 11.24 (7.5–22) ^	90.00 vs. 100.00	7 vs. 10 *	Likert: 4	1
**EVAR**							
Desender (2013) [23] (9 MDs) ^f^	10 **	32 (IQR 24–41) vs. 43 (IQR 39–60) *	13 (IQR 11–16) vs. 10 (IQR 8–18)	80 (IQR 75–97) vs. 80 (IQR 61–92)	5 (IQR 4–8.5) vs. 6 (IQR 4.5–7)	Likert: 4 (IQR 3–5)	1
Pakeliani (2019) [24] (1 MD) ^g^	10	54 ± 14 (37–80) vs. 69 ± 16 (45–90) +	N/A	N/A	N/A	N/A	1
**TEVAR**							
Desender (2017) [25] (2 MDs)	2	Sim. time for 1 patient: 16	N/A	N/A	N/A	N/A	1

CAS = carotid artery stenting; EVAR = endovascular aortic aneurism repair; TEVAR = endovascular thoracic aortic repair; N/A = not available; IQR = interquartile ranges; GRS = global rating scale; MD = Medical Doctor. All values are medians (range) unless otherwise stated. Metrics compare simulation vs. real case. * Statistically significant P-value (*p* < 0.05). ^ Range not stated, but manually read from a figure. + Mean ± standard deviation (range). ** One patient excluded from analysis. ^d^ Surgeons had previous carotid stent experience, mean of 51 (range 13 to 80) with a mean of 16 (range 6 to 20) over the previous year. ^e^ All were defined as experienced practitioners in CAS, meaning that all had performed more than 50 CAS procedures in total. ^f^ The lead interventionalists had performed more than 500 endovascular procedures, and 7/9 had performed at least 50 EVARs as the primary operator. ^g^ Performed more than 50 cases.

**Table 3:** Studies with a control group on the Medical Education Research Study Quality Instrument (MERSQI) scale.

Study	MERSQI
Wooster (2018) [17]	12.5
Desender (2016/2017) [13,16]	15
Våpenstad (2020) [18]	13

**Table 4:** Feasibility studies on the MERSQI scale.

Study	MERSQI
Hislop (2009) [19]	9
Roguin (2009) [20]	6.5
Willaert (2010) [21]	8
Willaert (2012) [22]	10
Desender (2013) [23]	10.5
Pakeliani (2019) [24]	9.5
Desender (2017) [25]	5
